# Supplementary material for: Nitrogen and Nod factor signaling determine Lotus japonicus root exudate composition and bacterial assembly
Source: Nat Commun. 2024 Apr 23;15:3436. doi: 10.1038/s41467-024-47752-0 (PMC11039659; doi:10.1038/s41467-024-47752-0)
Supplement: Supplementary file 3 — Description of Additional Supplementary Files [file 41467_2024_47752_MOESM3_ESM.pdf]

Description of Additional Supplementary Files:

Supplementary Data 1: Relative abundances (RA) of ASVs enriched in different genotypes and growth conditions. This table contains detailed information complementary to Figure 3 and Supplementary Figure 3. Abundant ASVs (RA > 0.3% in Gifu) are listed in the first column of each sub-table. The following columns show the RA of the ASVs in soil, Gifu, nfre, chit5, and nfr5, the comparison of RA between genotypes, and the taxonomy information of each ASV. Comparison pairs are shown as "Gifu_vs._soil", "nfre_vs._Gifu", "chit5_vs._Gifu", and "nfr5_vs._Gifu" implying the first item compared to the second item. The statistically enriched or depleted ASVs between comparison pairs are marked as "UP" and "DOWN", respectively. The information is shown separately in four sub-tables according to compartments (root, rhizosphere) and soil conditions (unfertilized, 10 mM KNO3-supplemented).

Supplementary Data 2: Ratio of relative abundances (RAs) in the root compartment in different genotypes grown in agricultural soil experiment. This table contains detailed information complementary to Supplementary Figure 4 and Supplementary Figure 5. The information is shown separately in two sub-tables according to the soil conditions (unfertilized, 10 mM KNO3-supplemented).

Supplementary Data 3: Differentially abundant ASVs in different genotypes, compartments when grown in unfertilized versus 10 mM KNO3-supplied soil conditions. This table contains detailed information complementary to Supplementary Figures 6-9. The statistically significant differential abundant ASVs (|logFC| > 1.5) in each genotype (Gifu, nfre, chit5, and nfr5) and compartment (rhizosphere and root) between unfertilized and 10 mM KNO3-supplemented soil conditions are listed in the sub-tables.

Supplementary Data 4: The node information of the cooccurrence network. The taxonomy, modularity class, correlation degree, betweenness centrality, clustering coefficient, closeness centrality, and eccentricity are presented for each node in the cooccurrence network analysis. The information is shown separately in six sub-tables for the three nutritional statuses (starved, symbiotic, inorganic) of Lotus in the two compartments (rhizosphere and root).

Supplementary Data 5: The identified predictor genus and prediction accuracy for rhizosphere and root. Sub-table "rhizosphere_predictor" listed the predictor genus in the rhizosphere; Sub-table "root_predictor" listed the predictor genus in the root; Sub-table "accuracy" listed the prediction accuracy of the selected genus by three machine learning models.

Supplementary Data 6: List of LjSphere isolates used in SynComs for reconstitution experiments. The isolate number with the corresponding ASV number and taxonomic information is shown in the table.

Supplementary Data 7: The enrichment and intensity of metabolites. The identified chemical class or chemical features in the four nutritional statuses (starved, symbiotic, inorganic, and both inorganic and symbiotic) of Lotus are listed in the two sub-tables “state_associated_class” and “state_associated_features”. The annotation, median value of intensity, and state category of listed chemicals are shown in the table. The result of statistical comparisons of the chemical intensities in the four nutritional statues is shown. “0” means no significant difference; “1” means significant enriched; “-1” means significant depleted.
